# Retention of hybrid-abutment-crowns with offset implant placement: influence of Crown materials and Ti-base height

**DOI:** 10.1186/s12903-023-03490-1

**Published:** 2023-10-24

**Authors:** Ahmed Alseddiek, Walid Al-Zordk, Ahmed Attia

**Affiliations:** Department of Fixed Prosthodontics, Faculty of Dentistry, Mansoura University, El Gomhouria St, P.O. Box 35516, Mansoura, Dakahlia Governorate Egypt

**Keywords:** Implant, Retention, Titanium base, Ceramics, CAD/CAM

## Abstract

**Background:**

The purpose of the current study was to assess the impact of three esthetic CAD/CAM material, titanium base height and their interaction on the retention strength of a hybrid-abutment-crown.

**Methods:**

A total of 42 hybrid-abutment crowns with identical external geometries were designed in CAD software to fit two different Ti-Base abutment heights (n = 42/abutment height): either short (S) with 4 mm (n = 21) or long (L) 7 mm (n = 21) height. Each main group was divided into 3 subgroups (n = 7), according to esthetic crown material, Zirconia (Z), Lithium disilicate (L) and Hybrid ceramic (V). A universal primer and an adhesive resin cement were used for bonding according to the manufacturer instructions. Artificial aging in form of water storage (30 days), chewing simulation (50,000-cycles, 49 N, 1.67 Hz) and thermal cyclic (5000 cycles at 5–55 °C) were applied, specimens were pulled-out under tension load in (N) using a universal testing machine. Two and one-way ANOVA and Post Hoc Tukey test were used for statistical analysis.

**Results:**

Long lithium disilicate (LL) group showed the highest retention (738.7 ± 178.5) followed by short lithium disilicate (LS) group (688.6 ± 169.9). Meanwhile, short zirconia (ZS) showed the lowest retention strength (231.1 ± 86.9).

**Conclusion:**

CAD/CAM fabricated lithium disilicate hybrid-abutment-crown can be used instead of conventional crowns over implant abutment. Etchable ceramics are recommended as a material of choice for CAD/CAM fabricated hybrid-abutment-crowns instead of zirconia in terms of retention durability.

## Background

Patients and professionals alike are increasingly favoring implant-supported crowns. The main reason for this is due to their ability to conserve the natural dental tissues of neighboring teeth and their favorable prognosis, which is backed by significant scientific research documented in the literature, including comprehensive clinical trials. The enduring efficacy of these crowns is contingent upon the fundamental biological and mechanical characteristics of the prosthetic superstructure [1]. The effectiveness of this treatment approach is not solely tied to the achievement of successful osseointegration; it is also closely linked to the corresponding aesthetic of superstructure [2].

Prosthodontic research primarily concentrates on enhancing materials to achieve improved biomechanical and aesthetic properties. The utilization of CAD/CAM technology has made the fabrication of implant-supported restorations more convenient and has decreased the complex laboratory procedures [3]. Ideal positioning of dental implants, sometimes, cannot be attained due to either anatomical limitations, such as the proximity of the maxillary sinus or mandibular canal to the implant in molar area, or a presence of a bony defect. Therefore the implant being placed in an offset configuration relative to the prosthetic unit [4]. Furthermore, positioning dental implants in an offset manner can potentially enhance the aesthetics of the restoration by reducing the emergence profile and simplifying dental hygiene in the posterior mandibular area. Nevertheless, there are apprehensions regarding the biomechanics of offset implants and the distribution of stress in the surrounding tissue due to the application of non-axial forces on these implants [5]. Hence, it is crucial to verify whether stress distribution occurs safely around the offset implants [6].

Implant abutments can be classified into two groups: ready-made and custom-made. Nevertheless, in many instances, the requirement for customization of the emerging profile to achieve improved biological and aesthetic results, facilitate retrieval, accommodate restricted inter-occlusal distance, and address challenges associated with excess cement removal necessitates the utilization of abutments that are specifically tailored to individual patients [7]. Ensuring the appropriate selection of the abutment holds significant importance in achieving both mechanical stability and aesthetically pleasing outcomes [8].

Prefabricated abutments present various advantages due to their uncomplicated application and cost-effectiveness. Nevertheless, these abutments frequently lack the necessary accurate form and shape, with a primary issue being the diameter of the platform and the corresponding soft tissue shape [9]. Custom abutments become necessary in specific clinical scenarios, such as instances where the implant manufacturers do not provide the required collar height, the need to replicate the original tooth’s cross-sectional profile to achieve an optimal emergence profile, and inadequate crown height space for the eventual restoration [10]. The utilization of titanium bases (Ti-bases) as attachments for restorations supported by dental implants is experiencing a notable increase in popularity, particularly due to their integration into the recently developed digital workflow. The use of prefabricated components facilitates an exact positioning and establishes a durable attachment between the implant and abutment, comparable to the level of achievement observed with regular titanium abutments [11].

Another key benefit of using the Ti-base is its adaptability in the construction process of restorations. It can be created through a fully analog method using traditional impressions and laboratory techniques. Alternatively, it can be entirely designed and manufactured digitally, starting with optical scanning of the scan body and progressing to CAD/CAM production of the abutment and prosthesis. The majority of CAD/CAM solutions continue to expand their database library for the fabrication of restorations on Ti-bases [12]. When a Ti-base is paired with a ceramic meso-structure or a fully contoured prosthesis (Hybrid-abutment-crown), it enables the conversion of a conventional element into a tailored and customized product [13]. The ceramic superstructure’s ability to be customized optimizes the soft tissue appearance and promotes improved adaptability to the adjacent soft tissue [14].

A titanium base can be externally bonded to a ceramic abutment. Following this, a ceramic crown is fixed either outside the mouth or within the mouth using adhesive cementation. This particular abutment configuration is commonly referred to as a hybrid abutment, as it merges the aesthetic benefits of ceramic materials with the mechanical durability offered by a metal implant-abutment attachment [15]. Nonetheless, the efficacy of the titanium base approach may rely on the durability of the bonded contact between the Ti-base and the ceramic components [16, 17].

Zirconia has exhibited favorable results in terms of fracture resistance when employed as abutments on titanium bases. The fracture strength of zirconia abutments in this particular arrangement demonstrates a significant increase in comparison to both pure zirconia abutments and two-piece zirconia abutments when utilized for single-tooth restorations in the anterior area [18].

The impressive mechanical properties and translucency of lithium disilicate ceramic have led to its use over Ti-bases, either as an abutment or an abutment crown. This integration can be accomplished through heat-pressing or CAD/CAM techniques. Ready-made lithium disilicate blanks with Ti-base connection geometry are accessible now for most implant systems. It’s believed that bonding lithium disilicate to Ti-base is simpler than with zirconia [19].

There has been a recent development of hybrid dental restorative materials that incorporate diverse nanostructures. These advancements aim to enhance the physical and biological properties of materials utilized in various areas of dentistry [20]. The novel polymer-infiltrated ceramic material melds the characteristics of both ceramics and polymers. It comprises a mixed structure featuring dual interwoven networks: one primarily ceramic and the other strengthening resin. This combination results in a double network hybrid ceramic material [21].

There has been limited research conducted on the cementation procedures for Ti-base abutments [22]. Previous studies evaluated the influence of CAD/CAM abutment height and type of cement on the retention of zirconia crowns and concluded that while the height of Ti-base abutments has shown no impact on the retentiveness of zirconia superstructures, it is noteworthy that resin-based cements have consistently demonstrated significantly superior retention compared to both glass ionomer and temporary cements [23]. Previous studies conducted by other researchers have indicated that a greater height-to-width ratio or taller axial walls of the abutment positively influence uniaxial testing values due to an increased contact area [24].

## Hypothesis

**The null hypotheses for this study are as follows**:

1. There is no statistically significant difference in the retention of hybrid-abutment-crowns that are bonded to titanium bases with varying heights.
2. The retention of hybrid abutment crowns is not influenced by the type of definitive prosthetic materials used.

## Methods

This study was approved by the ethical committee at Faculty of Dentistry, Mansoura University with reference number (A09030821).

The materials utilized in this study are presented in Table 1.

**Table 1:** Shows the materials used in this study

Materials	Product name	LOT number	Main composition	Manufacture
**Zirconia**	Ceramill Zolid HT + white	2,007,001	Main component: zirconium oxide (ZrO2) more than 99% by weight. Other contents: Aluminum oxide 0.25 wt% Yttrium oxide (Y_2_O_3_) 5.2wt%.	Amann Girrbach, Pforzheim, Germany
**Lithium disilicate glass ceramic**	IPS e.max CAD	Z02FXV	Main component: Lithium disilicate (SiO2) Other contents: Li2O, K2O, MgO, Al2O3, P2O5.	Ivoclar-Vivadent, Schaan/Liechtenstein
**Hybrid Ceramic**	VITA ENAMIC	91,020	Polymer-infiltrated-feldspatic ceramic-network material (UDMA, TEGDMA) with 86 wt% ceramic (Si02, Al203, Na2O, K20, B203, CaO, TiO2, colouring oxides)	VITA Zahnfabrik, Bad Säckingen, Germany
**Fixture**	V Plus Implant 4.2*13		Main component: commercially pure titanium grade 4.	Vitronex, Milano, Italy
**Titanium base abutment**	TBASE-MPI		Main component: commercially pure titanium grade 5.	Vitronex, Milano, Italy
**Hydrofluoric acid**	Porcelain etchant	2,300,000,134	9.5% Buffered Hydrofluoric acid gel	Bisco, Anaheim, CA, USA
**Universal primer**	Monoband plus	Z039MX	Alcohol, sulfide dimethacrylate, MDP, gamma-MPTS	Ivoclar-Vivadent, Schaan/Liechtenstein
**Resin cement**	Multilink hybrid-abutment cement	Z03NZG	Dimethacrylate, HEMA, fillers (barium glass, ytterbium trifluoride, spheroid mixed oxides, titanium dioxide)	Ivoclar-Vivadent, Schaan/Liechtenstein

The calculation of the sample size was performed using G* (version 3.0.10). In order to discern a 5% disparity with an effect size of 1.72, it was necessary to have a sample size of 7 specimens for each group.

**Fig. 1:**
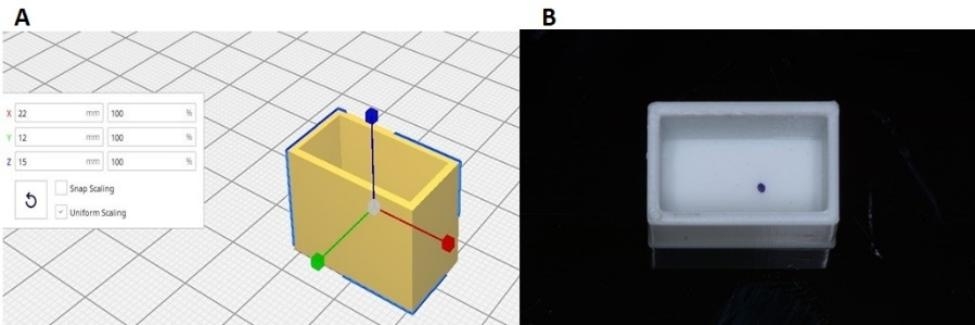
Shows the design and 3D-printed acrylic box. **(A)** The designing **(B)** The 3D-printed model.

**Fig. 2:**
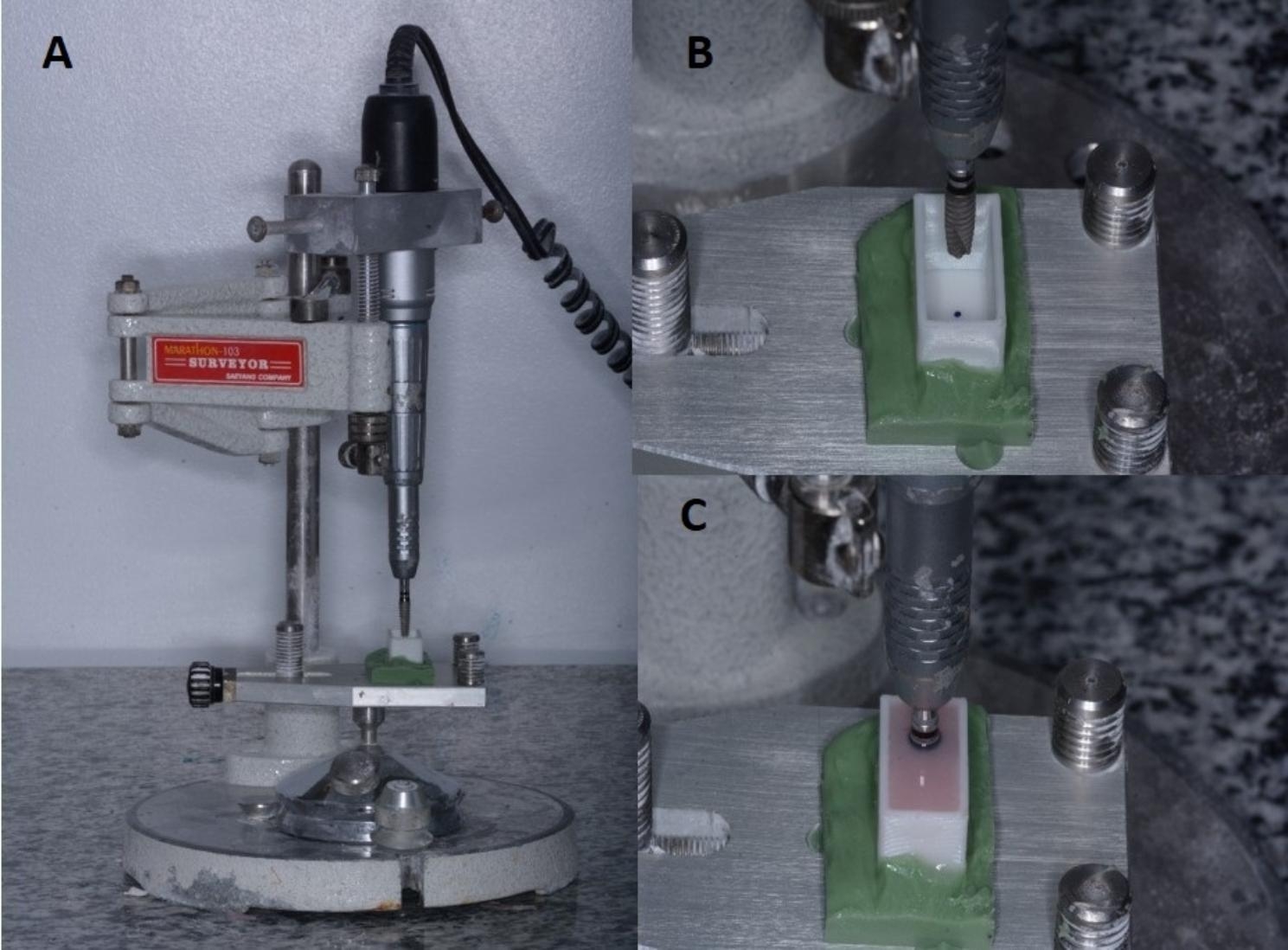
Shows the implant fixture being held in the surveyor and fixed in the same position for standardization and acrylic poured around it

A total of 42 3D-printed PMMA (NextDent, AV Soesterberg, Netherland) boxes with dimensions of 22 × 12 × 15 mm were designed and printed using a 3D printer (Mogassam, Cairo, Egypt). A marker was used to determine an offset point which is 5 mm distal to the center of the block to mimic distally positioned implant. (Fig. 1) To ensure a consistent alignment, Ti-base abutments were torqued onto the fixtures and securely positioned in a surveyor (Marathon-103, Saeyang, Daegu, Korea). Subsequently, self-cured acrylic resin (Acrostone, Alex, Egypt) was mixed and poured into 3D-printed PMMA boxes, with the implants’ long axis aligned to the boxes. This method was employed to establish a standardized vertical orientation of both the implants and Ti-base abutments for the subsequent pull-out testing. (Fig. 2)

### Scanning, designing, and milling procedures

**Fig. 3:**
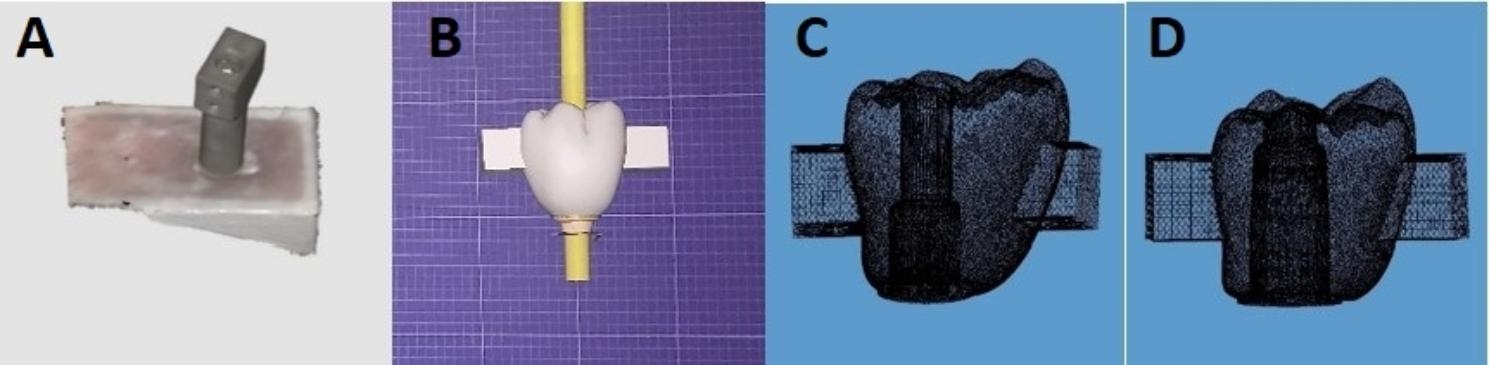
Shows the scanning and different crown designs according to Ti-base height

**Fig. 4:**
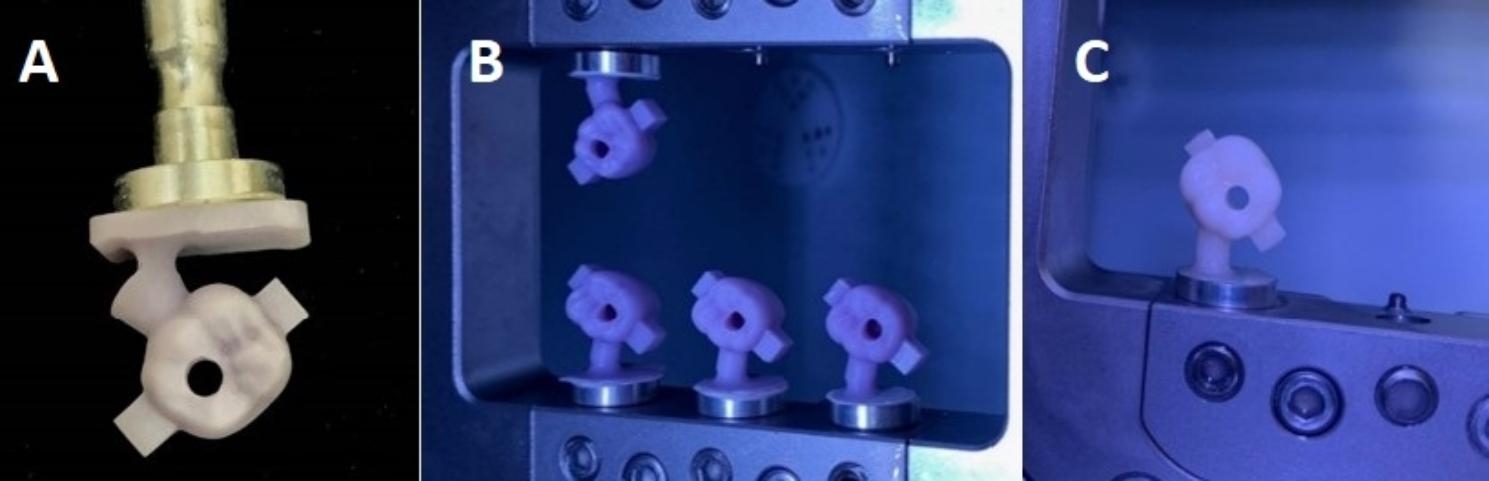
Shows the milled crowns with the offset distally placed

A scan body was attached to the implant and scanned using Medit i700 intraoral scanner (Medit, Seoul, South Korea). After that, a series of 42 crowns featuring uniform external geometries were meticulously crafted using a CAD software (Exocad dental CAD GmbH, 64,293-Darmstadt, Germany) to accommodate two distinct Ti-base (MPI-TBASE, Vitronex, Milano, Italy) abutment heights (n = 42): with two different heights 4 mm short (S) (N = 21) and 7 mm long (L) (N = 21). At the mesial and distal surfaces of the each crown, projections were designed 3 mm below the occlusal surface with dimensions of 3.5 × 4 × 4 mm (Fig. 3) with the objective of accommodating stainless steel wires for the purpose of conducting pull-out tests. The screw channels were located in a distal position. During CAD-CAM fabrication of all crowns. The offset was placed 5 mm distally (Fig. 4) to mimic the mesially-positioned implant. This was standardized to ensure that the implant is placed in the same position in all specimens.

In addition, the main group was further subdivided into two subgroups (n = 7) based on the three ceramic materials employed for the manufacturing of the crowns, namely zirconia (Z), lithium disilicate (L), and Vita Enamic (V).

Three distinct types of machinable ceramics were utilized in this study, namely zirconia (ceramill zolid HT+, Amann Girrbach, Pforzheim, Germany), lithium disilicate (IPS e.max CAD, Ivoclar-Vivadent, Schaan/Liechtenstein), and hybrid ceramic (Vita Enamic, VITA Zahnfabrik, Bad Säckingen, Germany). These ceramics were divided into six groups, denoted as ZL, ZS, LL, LS, VL, and VS, with a total sample size of seven (N = 7). The milling procedure was conducted using a 5-axis milling machine known as the Ceramill Motion 2 (Amann Girrbach AG, Herrschaftswiesen, Germany). (Fig. 4)

### Surface treatment of different crown materials

**Zirconia crowns (ZS, ZL)** intaglio surfaces were conditioned by air-abrasion using 50 μm Al_2_O_3_ Particles at an applied pressure of 2 bar for 15 s, with a 2 cm step-over distance. The surfaces were then irrigated with water for 60 s and ultra-sonically cleaned (MCS digital ultrasonic cleaner) in 95% isopropanol for 3 min. The surfaces were then thoroughly dried with a compressed dry air stream for 10 s [25].

### IPS e.max CAD (LS, LL) and Vita enamic (VS, VL)

The intaglio surfaces were conditioned with 9.5% buffered HF acid (Porcelain etchant, Bisco, Anaheim, CA, USA) for 20 s. The surfaces were then irrigated with water for 60 s and ultra-sonically cleaned (MCS digital ultrasonic cleaner) in 95% isopropanol for 3 min. The surfaces were then thoroughly dried with a compressed dry air stream for duration of 10 s.

### Primer application

Following dryness specimens of the three test groups were conditioned with primer as follow: a thin layer of a universal primer (Monobond plus, Ivoclar Vivadent, Schaan/Liechtenstein) was applied using a micro brush and allowed to sit for 60 s. Any excess primer was removed by blowing it away with a strong stream of air [26, 27].

The Ti-bases underwent air abrasion with 50 μm Al_2_O_3_ particles at a pressure of 2 bar for 15 s, with a 5-mm step-over distance. Following this, the specimens underwent a cleaning process utilizing an ultrasonic cleaner (CD-4820 digital ultrasonic cleaner, Codyson, China) with 95% isopropanol solution for a duration of 3 min, after which they were subjected to a drying procedure. Subsequently, the Ti-bases were treated with a universal primer (Monobond plus). The primer was gently rubbed onto the Ti-bases for a duration of 20 s, followed by a reaction period of 60 s. Finally, a moderate stream of air was used to dry the Ti-bases [28].

### Bonding

The screw channels were sealed by placing pieces of Teflon tape inside them. Bonding was carried out using adhesive resin cement (Multilink Hybrid-abutment, Ivoclar Vivadent Schaan/Liechtenstein) following the manufacturer’s instructions. To secure the specimens, a specially designed device with a lever system was employed to apply a 5 kg load on the occlusal crown-abutment assembly during the bonding process. The bonded component was left under this static load for 5 min. Light curing was performed from four different directions, each lasting for 20 s. Finally, any excess cement was removed, and the surface was finished and polished [29, 30].

### Artificial aging

All specimens were stored in a 37 °C water bath for 30 days, starting one hour after cementation. Samples were stored in containers made of waterproof plastic material. Following that, fatigue tests under cyclic loading were performed utilizing a CS-4 chewing simulator machine (manufactured by SD Mechatronik GMBH, located in Feldkirchen, Germany). The specimens were subjected to 5000 thermal cycles (ranging from 5 to 55 °C, with a 30-second dwell time at each temperature extreme) and 50,000 repeated cyclic loads with a weight of 49 N and a loading frequency of 1.7 Hz to simulate 2.5 months of normal mastication [31].

### Pull-off retention test

**Fig. 5:**
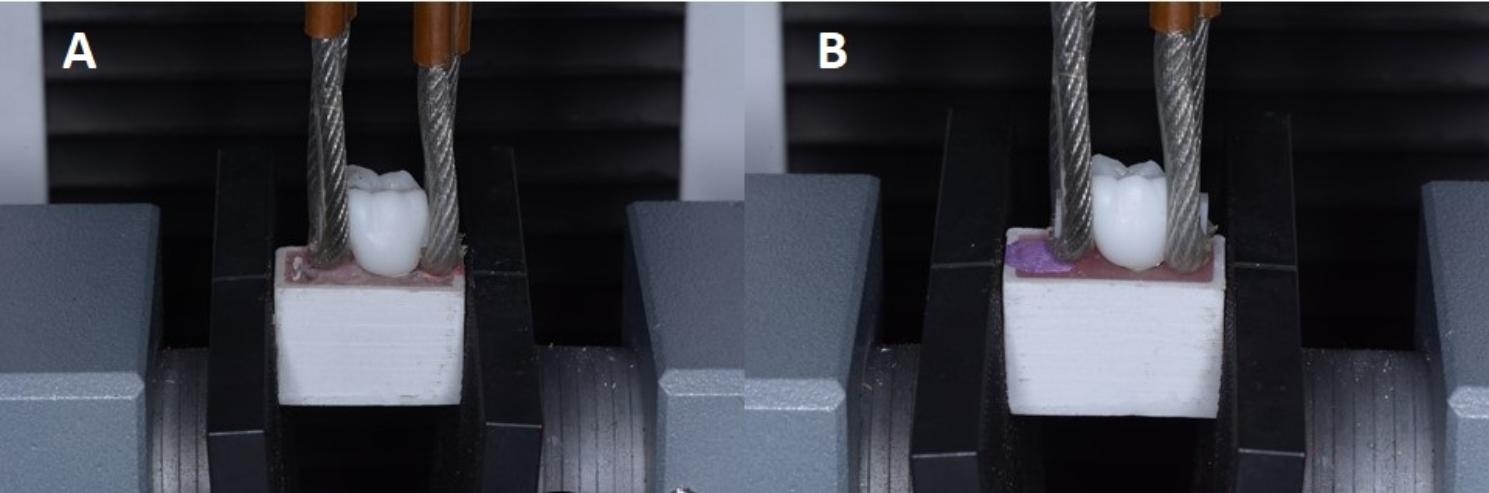
Shows specimen fixed in the universal testing machine for pull-out retention test

Two stainless stell wires of around 1.8 mm were wrapped around the 2 arms below occlusal surfaces of each crown. These wires were then secured within a device that was positioned right beneath the load cell. The pull-off testing procedure was conducted using a universal testing equipment (Instron, United States). The test was carried out at a crosshead speed of 1 mm/min until the occurrence of crown dislocation and load decrease. Pulling force in (N) was measured for each specimen and tabulated for statistical analysis. (Fig. 5)

### Statistical analysis

The statistical analyses were performed using the Social Package for Statistical Science (SPSS) software, specifically version 25.0. The analysis utilized a two-way analysis of variance (ANOVA), followed by sequential one-way ANOVAs conducted at each level of the study. Subsequently, Post Hoc Tukey (HSD) tests were employed. Statistical significance was established by considering a p-value below 0.05 (P < 0.05).

## Results

The collected data were systematically arranged, coded, and analyzed.

The mean retentive force (N) of all test groups was analyzed using a 2-factor ANOVA model (Table 2). The factors considered in the analysis were crown materials, Ti-base height, and their interactions. The ANOVA F-test yielded a significant result (P < 0.0001), suggesting that there is a significant difference in the mean retention force among at least one of the factors. The crown material (P < 0.0001) and height of the Ti-base (P < 0.0001) demonstrated statistical significance. However their interaction was not significant (p = 0.059).

Serial (ANOVAs) at each level of the study was conducted. Crown material was statistically significant P (P < 0.0001) (Table 3). Also the Ti-base height was statistically significant, (P = 0.0007) (Table 4).

The Post Hoc Tukey (HSD) test was utilized for conducting paired comparisons among each two test groups (Table 5). Considering crown materials with the same Ti-base height, a statistically significant difference was observed between the following test groups: (ZS - LS, P < 0.0001), (ZL - LL, P = 0.001), and (LS -VS, P < 0.0001).

**Fig. 6:**
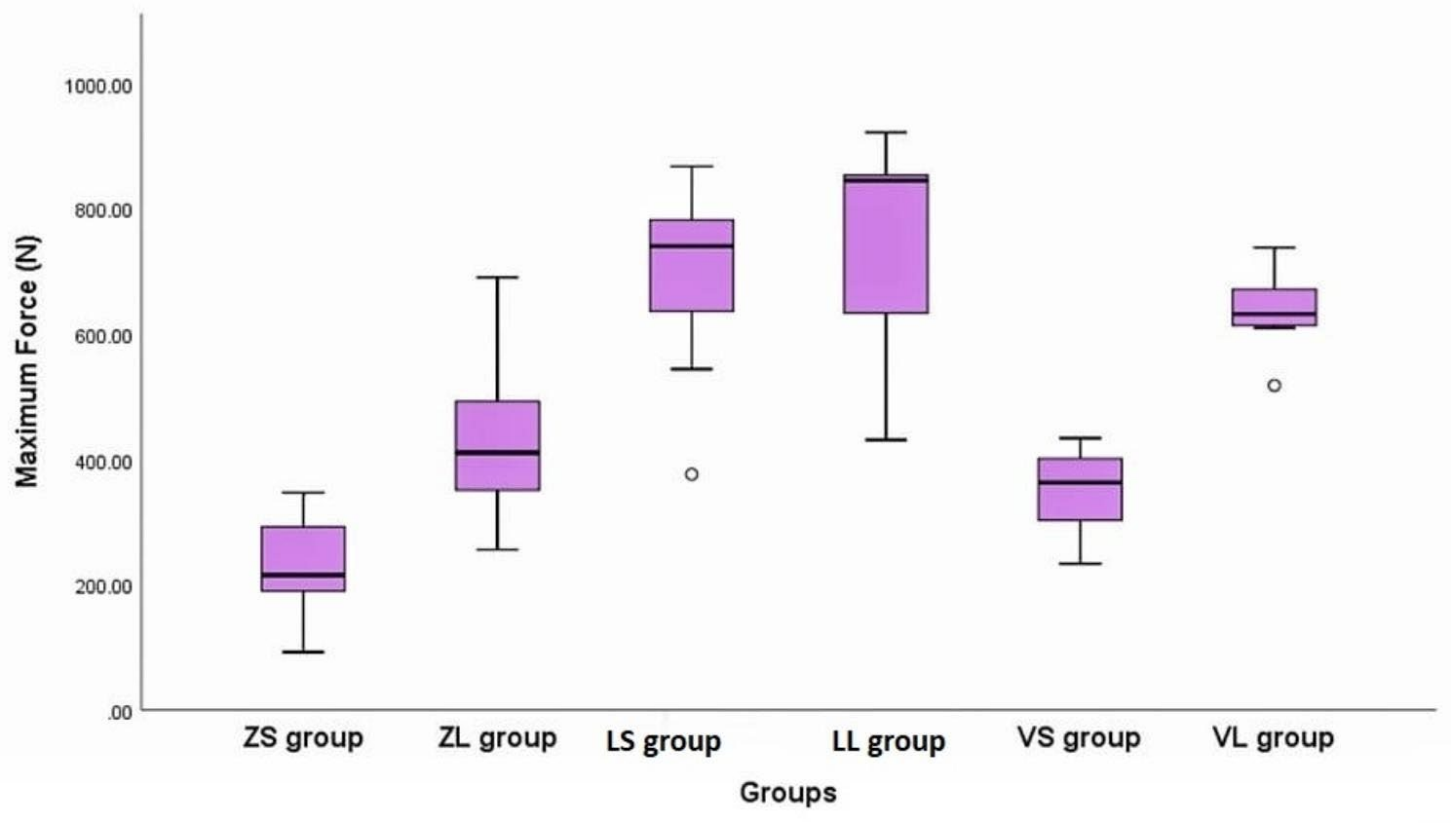
Box plots illustrate the mean retention force values of tested groups in (N)

Similarly, when considering Ti-base height within the same crown material, statistically significant disparities were recorded among two test groups (VS - VL, P = 0.002). Nevertheless, the mean retentive force of remaining test groups showed no significant difference (P > 0.05). The previous results were confirmed using Box-plots test (Fig. 6).

**Table 2:** Showing two-way ANOVA test for crown materials and Ti-base height and their interaction

Source	Type II Sum of Squares	df	Mean Square	F	*p*	Power ^b^
Model	12521135.377^a^	6	2086855.896	125.037	**0.000** ^*^	1.000
Crown materials	1023410.173	2	511705.087	30.660	**0.000** ^*^	1.000
Ti-base height	343158.404	1	343158.404	20.561	**0.000** ^*^	0.993
Crown × Base	102289.585	2	51144.792	3.064	0.059	0.556
Error	600834.823	36	16689.856			
Total	13121970.200	42				

^a^ R Squared = 0.954 (Adjusted R Squared = 0.947),

^b^ Computed using alpha = 0.05,

^*^ Significant difference (*p* < 0.05).

**Table 3:** Showing One-Way ANOVA considering crown materials

	Sum of Squares	df	Mean Square	F	Sig.
Between Groups	1023410.173	2	511705.087	19.074	0.000
Within Groups	1046282.811	39	26827.764		
Total	2069692.985	41			

**Table 4:** Showing One-Way ANOVA considering Ti-base height

	Sum of Squares	df	Mean Square	F	Sig.
Between Groups	343158.404	1	343158.404	7.950	0.007
Within Groups	1726534.581	40	43163.365		
Total	2069692.985	41			

**Table 5:** Showing means ± SD retentive force of tested groups in (N): Post Hoc Tukey HSD test (p ≤ 0.05)

Group	Group-1 (ZS)	Group-2 (ZL)	Group-3 (LS)	Group-4 (LL)	Group-5 (VS)	Group-6 (VL)	N	Mean ± SD
Group-1 (ZS)	—	0.058	**0.000** ^*^	**0.000** ^*^	0.54	**0.000** ^*^	7	231.1 ± 87
Group-2 (ZL)		—	**0.009** ^*^	**0.001** ^*^	0.81	0.06	7	434.7 ± 147.5
Group-3 (LS)			—	0.98	**0.000** ^*^	0.97	7	688.6 ± 169.9
Group-4 (LL)				—	**0.000** ^*^	0.68	7	738.7 ± 178.5
Group-5 (VS)					—	**0.002** ^*^	7	348.1 ± 73.4
Group-6 (VL)						—	7	636.7 ± 68.7

^*^ Significant difference (*p* < 0.05). (A cell represents a pairwise *p-value*.)

ZS = short zirconia ZL = long zirconia

LS = short lithium disilicate LL = long lithium disilicate

VS = short vita enamic VL = long vita enamic

(*) indicating statistically significant difference

## Discussion

The purpose of this in vitro study was to evaluate the influence of three machinable ceramics and height of Ti-bases on the retentive strength of the hybrid-abutment-crowns after artificial aging.

The hypothesis of the study was that neither the type of hybrid-abutment crown materials nor the height of Ti-bases could influence retention of CAD/CAM fabricated hybrid-abutment-crown. Nevertheless, the findings of this study contradicted both hypotheses under examination. Because both factors influenced the retentive force of the hybrid abutment crowns.

To replicate conditions inside the mouth, the specimens were immersed in water for 30 days after the cementation process, after which they underwent 50,000 cycles of cyclic loading fatigue along with 5,000 thermal cycles. It’s worth noting that these aging procedures did not result in the failure of any of the restorations.

Retention of 7 mm (long) Ti-base was significantly higher than 4 mm (short) Ti-base regardless of the crown material. Also, crown materials affected retention of hybrid-abutment-crown at same the height of Ti-base.

Retention of fixed dental prosthesis depend on total surface area of the abutment, in this study abutments have the same diameter, surface area of the abutment could by calculated by multiplying the diameter by the height of the abutment, therefore the longer abutment has more surface area than shorter one, consequently, reported higher retention regardless of the prosthetic materials used for the definitive restoration. This fact was reported by other researchers **Bernal et al. 2003** [24].

In this study mean retentive force (N) for zirconia crowns bonded to short titanium bases (ZS group) was 231.1 ± 87 N, while the mean retention force (N) for zirconia crowns bonded to long titanium bases (ZL group) was 434.7 ± 147.5 N, with significant difference between them. Considering retention of Vita enamic crowns bonded to short titanium bases (VS group) was 348.1 ± 73.4 N, compared to 636.7 ± 68.7 N, over long titanium bases with a significant difference between them. Also, the mean retentive force (N) considering IPS e.max CAD crowns over short Ti-bases (LS group) was 688.6 ± 169.9 N and (LL group) was 738.7 ± 178.5 N. This clearly proves that the Ti-base height significantly affects the retention regardless of the type of crown material used.

This came to an agreement with **Silva et al.** [32] who conducted a study to assess the effect of two distinct heights of Ti-bases (4 and 2.5 mm) on the retention of zirconia crowns using the pull-out test. The researchers observed a noteworthy effect of height of the abutment on the retention of the crowns.

Moreover, in other researches, **Zahoui et al.** [21] and **Abbo et al.** [33] concluded that the height of the Ti-base abutment had an impact on the tensile strength of CAD/CAM zirconia crowns supported by dental implants. Specifically, longer abutments exhibited higher retentive strength compared to shorter abutments.

Considering the three ceramic materials used it is proved that ceramic materials significantly affected retentive strength with the least retention was recorded for ZS group while the highest retention was recorded for LL group.

Many factors could influence retention of zirconia to titanium such as surface treatment including air abrasion, silica coating, primer application to enhance chemical bonding and type of cement. But all these factors were fixed through all test groups so that only the height of Ti-bases was the determining factor affecting the retention.

The longevity of the bond between the abutment and Ti-base is significantly influenced by the specific type of ceramic material utilized. According to **Dhesi et al.** [34], the type of ceramic and cement used significantly influences the bond strength values when bonding to a titanium basis. The findings of the study indicate that there is a higher average bond strength observed in lithium disilicate definitive restorations on a titanium base when combined with adhesive resin cement containing MDP, in comparison to zirconia restorations bonded to a titanium foundation using the same type of cement.

This investigation revealed significant disparities in the mean retentive force (N) of the different crowns over the same height of Ti-base. The mean retentive force (N) in lithium disilicate crowns over long titanium bases (LL group) was 738.7 ± 178.5 N and the mean maximum force (N) in Vita enamic crowns over long titanium bases (VL group) was 636.7 ± 68.7 N while the mean retentive force (N) for zirconia crowns over long titanium bases (ZL group) was 434.7 ± 147.5 N.

Different chemical composition of the three ceramic materials used might explain these results. IPS e.max CAD is lithium disilicate glass ceramic when it is etched with HF acid. Glass was partially dissolved creating micro surface pores, when the luting cement was applied to the treated intaglio surface micromechanical retention was formed. On the other hand, as stated by the manufacturer, Monobond Plus is a versatile primer comprising an alcohol solution that includes 3-methacryloxyprophyl-trimethoxysilane (MPS), phosphoric acid methacrylate, and sulphide methacrylate. As a result, it comprises the identical adhesive constituent (MPS) accountable for facilitating chemical adhesion with silica ceramics, in addition to the other two functional components (phosphoric acid methacrylate and sulphide methacrylate). This resulted in double chemical bonding to etched lithium disilicate glass ceramic. Although the same surface treatment was done for vita enamic restorations retentive strength was less than retentive strength recorded for IPS e.max CAD. This could be traced to variations in the chemical composition. According to the manufacturer, vita enamic is a hybrid ceramic with polymer-infiltrated-feldspathic ceramic-network material, it is clear from this composition that the disilicate component is nearly absent compared to IPS e.max CAD which is lithium disilicate glass ceramic. Consequently, HF acid etching was not effective for creating micro surface pores as in lithium disilicate glass ceramic, the net result was weak micromechanical bonding to vita enamic compared to IPS e.maxCAD. However chemical bonding due to Monobond plus universal primer was still effective through different adhesive components included in the chemical composition of the primer which created a durable chemical bonding to feldspathic and (UDMA, TEGDMA) components included in the composition of vita enamic.

Considering zirconia ceramics, airborne particle abrasion was used for condition the intaglio surfaces, which creating micro surface pores, removed surface impurities, improved surface tension, and wettability of the zirconia surface therefore a micromechanical bonding was achieved. However, chemical bonding was mainly due to two functional components (phosphoric acid methacrylate and sulphide methacrylate) component in the universal primer Monobond plus, because adhesive component (MPS), could create chemical bonding only to silica-based ceramics not oxide ceramics as zirconia. Therefore, the original weak chemical bond was obtained with zirconia compared to durable bond to lithium disilicate glass ceramic. Although few studies are available regarding the retentive strength of the same materials when bonded to titanium, different studies concluded that the retention of vita enamic and e.maxCAD was significantly higher than zirconia restorations when bonded to tooth structure [35, 36].

**Bjelopavlovic M et al.** [37] Studied different CAD/CAM ceramic crowns bonded to titanium implant and concluded that the ceramic material used has a Considerable impact on the retentive strength of the crowns with the highest retention force values (N) found in e.maxCAD group followed by Vita enamic group.

Similarly, a previous study by **Ongun et al.** [38] highlighted the increased bond strength seen in lithium disilicate ceramics, which can be due to their higher silica content and the enhanced chemical bonding between lithium disilicate crystals and silane coupling agents. Consequently, the researchers highly advocate the application of lithium disilicate ceramics in the fabrication of personalized hybrid implant abutments due to their exceptional high bond strength.

## Conclusion

Within the limits of this in vitro study, it is possible to say the following:

1. The retention of the three different hybrid-abutment crown types is greatly improved by the height of the abutment evaluated.
2. The type of ceramic restoration affects the retention strength of hybrid-abutment crowns with lithium disilicate exhibiting the highest retention.

### Limitation of the study

Although crown retention is a key aspect for long term durability of definitive restorations. Other factors such mechanical properties of the selected ceramic materials could influence durability of these restorations. Therefore fracture resistance should be considered during the selection of the crown material.

## Data Availability

The data sets used and/or analyzed during the current study are available from the corresponding author upon reasonable request.

## Abbreviations

HF: Hydrofluoric acid
CAD/CAM: Computer aided design; computer aided manufacture
3D: Three dimensional
SPSS: Social Package for Statistical Science
