# Correction: Retention of hybrid-abutment-crowns with offset implant placement: influence of Crown materials and Ti-base height

**DOI:** 10.1186/s12903-023-03843-w

**Published:** 2024-02-07

**Authors:** Ahmed Alseddiek, Walid Al-Zordk, Ahmed Attia

**Affiliations:** Department of Fixed Prosthodontics, Faculty of Dentistry, Mansoura University, El Gomhouria St, P.O. Box 35516, Mansoura, Dakahlia Governorate Egypt

**Correction: BMC Oral Health 23, 784 (2023)**

**https://doi.org/10.1186/s12903-023-03490-1**

In this article [1], the author’s name Walid Al-Zordk was incorrectly written as Walid Alzordok.

The original article has been corrected.
